# *BTNL2* Gene Polymorphism and Sarcoidosis Susceptibility: A Meta-Analysis

**DOI:** 10.1371/journal.pone.0122639

**Published:** 2015-04-07

**Authors:** Yihua Lin, Jia Wei, Lili Fan, Deyun Cheng

**Affiliations:** 1 Department of Respiratory Medicine, West China Hospital of Sichuan University, Chengdu, Sichuan, China; 2 Department of Respiratory Medicine, the First Affiliated Hospital of Xiamen University, Xiamen, Fujian, China; University of Birmingham, UNITED KINGDOM

## Abstract

**Background:**

Butyrophilin-like 2 (*BTNL2*) rs2076530 gene polymorphism has been implicated in susceptibility to sarcoidosis. However, results from previous studies are not consistent. To assess the association of *BTNL2* polymorphism and sarcoidosis susceptibility, a meta-analysis was performed.

**Methods:**

PubMed, Embase were searched for eligible case-control studies. Data were extracted and pooled odds ratios (OR) with 95% confidence intervals (CI) were calculated.

**Results:**

Ten studies involving a total of 3303 cases and 2514 controls were included in this meta-analysis. Combined data indicated that *BTNL2* rs2076530 polymorphism was associated with sarcoidosis susceptibility in allelic model (A vs. G, OR = 1.59, 95%CI: 1.47–1.72), dominant model (AA + AG vs. GG, OR = 2.10, 95%CI: 1.67–2.65), and recessive model (AA vs. AG + GG, OR = 1.93, 95%CI: 1.49–2.50).

**Conclusions:**

This meta-analysis indicates that *BTNL2* rs2076530 polymorphism contributes to the risk of sarcoidosis.

## Introduction

Sarcoidosis is a multisystemic inflammatory disease with unknown aetiology, characterized by the formation of noncaseating granulomas and an exaggerated cellular immune response due to increased inflammatory activity of macrophages and CD4 helper T cells[1,2]. Many studies show that genetic susceptibility and environmental factors contribute to disease development. Among multiple potential factors contributing to sarcoidosis, one of them is the polymorphism of butyrophilin-like 2 (*BTNL2*) gene.

*BTNL2*, a member of the immunoglobulin superfamily, reduces proliferation and cytokine production from activated T cells, suggesting a role of functional *BTNL2* as a negative costimulatory molecule involved in T-cell activation[3]. The G→A transition constituting rs2076530 (G16071A) leads to a premature truncation of the protein, thereby disrupting insertion in the cell membrane, which is a necessary process for downregulating activated T cells[4,5]. So the truncated *BTNL2* seems to be involved in sarcoidosis. Several studies have been undertaken to evaluate this potential relationship between *BTNL2* rs2076530 polymorphism and sarcoidosis susceptibility. However, results from different reports of different geographic areas are not consistent. Besides, many studies were small in size and, as such, may on their own lack sufficient statistical power to address this issue adequately. In an effort to clarify the association of *BTNL2* rs2076530 polymorphism and sarcoidosis susceptibility, we performed a meta-analysis on all the studies identified by systematic review of literatures.

## Methods

### Literature sources and search strategy

This meta-analysis followed the Preferred Reporting Items for Systematic Reviews and Meta-analysis (PRISMA) criteria[6]. Two investigators (Y.Lin, J.Wei) searched PubMed, Embase databases up to 4 August 2014 independently. Disagreements were resolved through discussion or adjudicated by a third author (D.Cheng). All eligible articles were screened, and their references were checked for other relevant studies. The search strategy was as follows: (“sarcoidosis” or “Schaumann Syndrome” or “Boeck Disease” or “Besnier Boeck Schaumann Syndrome”) and (“SNP” or “variant” or “polymorphism” or “mutation”) in combination with (“BTNL2” or “butyrophilin-like 2” or “rs2076530”). There was no restriction on languages.

Reports that fulfilled the following criteria were included: (1) evaluating the *BTNL2* rs2076530 polymorphism and sarcoidosis risks; (2) case-control designs on unrelated individuals; (3) odds ratios (OR) with 95% confidence intervals (CI) or sufficient data for their calculation were available; (4) the control subjects satisfied the Hardy-Weinberg equilibrium (HWE); (5) the diagnoses of sarcoidosis were consistent with the International Consensus Statement on Sarcoidosis[7,8] or verified by histology. And reports were excluded if any of the following conditions existing: (1) reviews, abstracts and studies with overlapping or repeated data, (2) data about allelic frequencies could not be obtained. In case of overlapping or repeated studies, the one with most subjects was chosen.

### Quality assessment

Two authors evaluated the study quality independently according to the Newcastle-Ottawa scale (NOS)[9], which assesses the quality of non-randomised studies on the basis of selection of participants, comparability of groups, and exposure assessment. Disagreement was settled as described above.

### Data extraction

The data were extracted by two investigators (Y.Lin, J.Wei) independently and a consensus was reached on all items. Any disagreement was resolved as described above. The following data was collected: first author, year, ethnicity, sample size, mean age, gender ratio, sarcoidosis criteria, source of controls, and genotype or allele distribution in cases and controls.

### Statistical analysis

The data were analyzed using Review Manager 5.2 (The Nordic Cochrane Centre, The Cochrane Collaboration, Copenhagen, Denmark) and STATA 12.0 software (Stata Corp LP, College Station, TX, USA). Pearson’s χ^2^ test was used to determine whether the observed frequencies of genotypes in control groups conformed to HWE. The strength of the associations between *BTNL2* rs2076530 polymorphism and sarcoidosis risk was evaluated using OR with 95% CI. The pooled ORs were calculated for allelic model (A *vs*. G), dominant model (AA + AG *vs*. GG) and recessive model (AA *vs*. AG + GG) respectively. Heterogeneity was calculated using the *Q*-test and *I* ^*2*^-statistics[10]. If the *P*-value of the *Q*-test was>0.10, the pooled OR was assessed in a fixed-effects model; otherwise, the random-effects model was applied[11]. *I* ^*2*^ values were used to quantify heterogeneity[12]. *Z*-test were used to determine the significance of the pooled OR, in which a *P*-value of <0.05 was considered statistically significant. Subgroup analyses were performed to evaluate the ethnic-specific effects. For the subgroup analysis by ethnicity, the study populations were stratified into three groups: Caucasians, Asians, and mixed population. We also performed sensitivity analysis by extracting a single study each time to check the stability of the results. Publication bias was assessed using Egger’s test, Begg’s test and funnel plot.

## Results

### Study inclusion and characteristics

**Fig 1:**
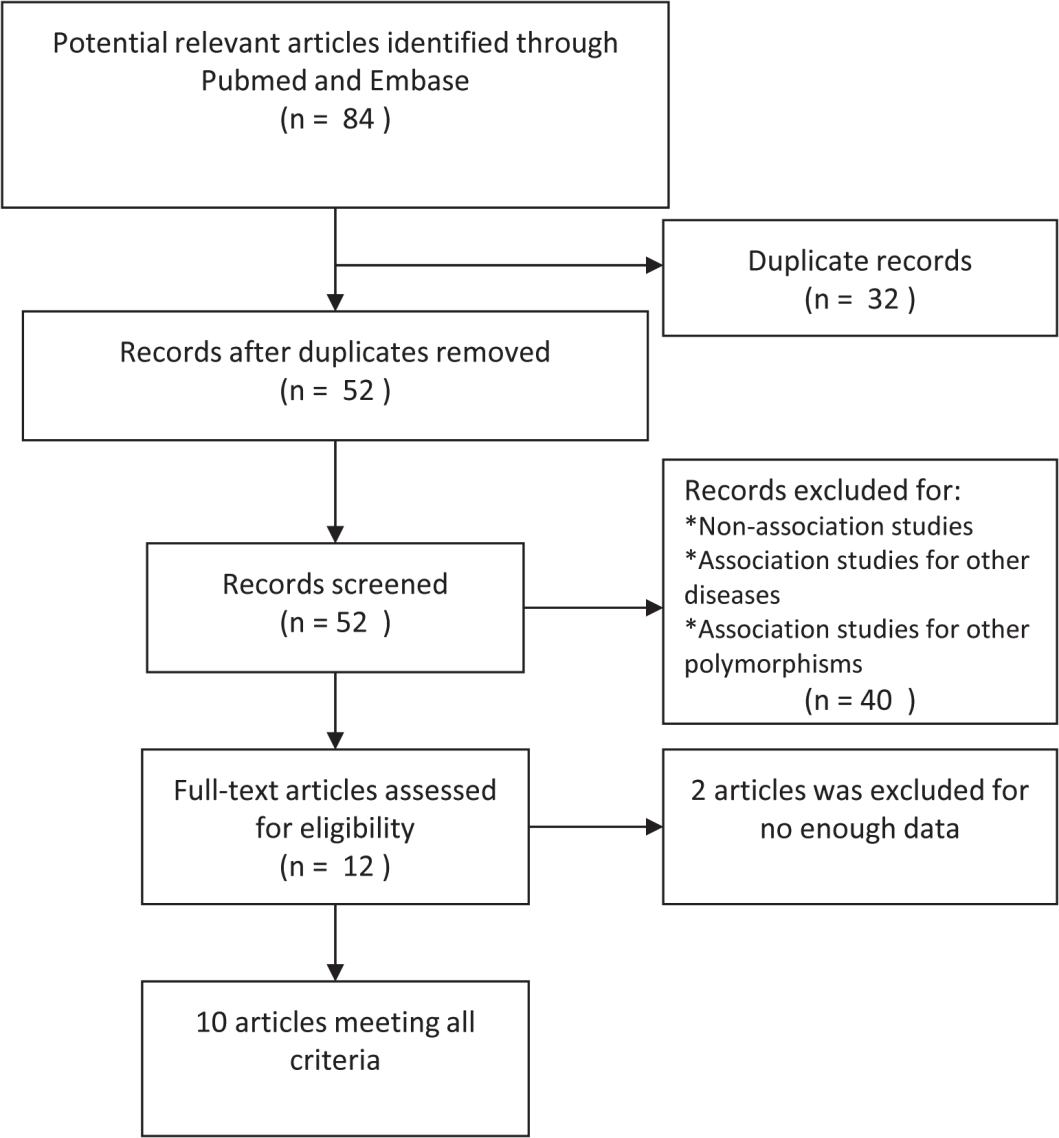
Flow of study indentification, inclusion and exclusion.

The procedure for including eligible studies is shown in Fig 1. A total of 84 articles were retrieved after initial search. Among them, thirty-two duplicate records were removed. After evaluating titles and abstracts, forty results were excluded for not relating to original study about association of *BTNL2* rs2076530 polymorphism and sarcoidosis susceptibility. After reading the full text, two articles were excluded for not enough data available. Finally, ten articles were included (the subjects of eight studies were Caucasion population[4,13,14,15,16,17,18,19], one study included Asian population[20] and one study involved mixed population[21]), containing 3303 cases and 2514 controls. In seven studies[4,13,15,17,18,19,20], the diagnoses of sarcoidosis were consistent with the International Consensus Statement on Sarcoidosis[7,8]. In the other three studies[14,16,–21], the diagnoses of sarcoidosis were verified by histology. Of the ten included studies, five articles[4,14,16,19,21] provided genotype distributions of *BTNL2* gene rs2076530 in sarcoidosis group (containing 1629 subjects) and control group (containing 1065 subjects). In the other five articles[13,15,17,18,19], only allele distributions could be obtained. It is noteworthy that the study by Rybicki et al[13] was performed in two ethnic populations (Caucasion and African American), but only the data of Caucasion population could be obtained through the article. We tried to contact the authors via email to get more information, but did not get any reply. The NOS scores of the including studies ranged from 7 to 9, indicating that the methodological quality was generally good. The general characteristics of all the including studies are listed in Table 1.

**Table 1:** The baseline characteristics of all study populations in the meta-analysis.

First author	Year	Country	Ethnicity	Score	Total sample size		Allele distributions	
					Cases	Controls	Cases		Controls	
							A	G	A	G
Rybicki BA	2005	USA	Caucasion	8	366	366	483	249	403	329
Valentonyte R	2005	German	Caucasion	8	904	427	1241	567	489	365
Li Y	2006	German	Caucasion	7	210	202	291	129	250	154
Spagnolo P	2007	UK and Netherlands	Caucasion	8	288	446	386	190	512	380
Milman N	2011	Denmark	Caucasion	9	87	113	129	45	126	100
Wijnen PA	2011	Netherlands	Caucasion	9	632	200	836	428	240	160
Morais A	2012	Portugal	Caucasion	9	151	150	197	105	167	133
Suzuku H	2012	Japan	Asian	8	237	287	355	119	355	219
Ozdemir M	2014	Turkey	Caucasion	7	375	271	59	47	48	56
Gaillot-Drevon M	2014	France	Mixed	7	53	52	512	238	295	247

### Quantitative analysis

**Fig 2:**
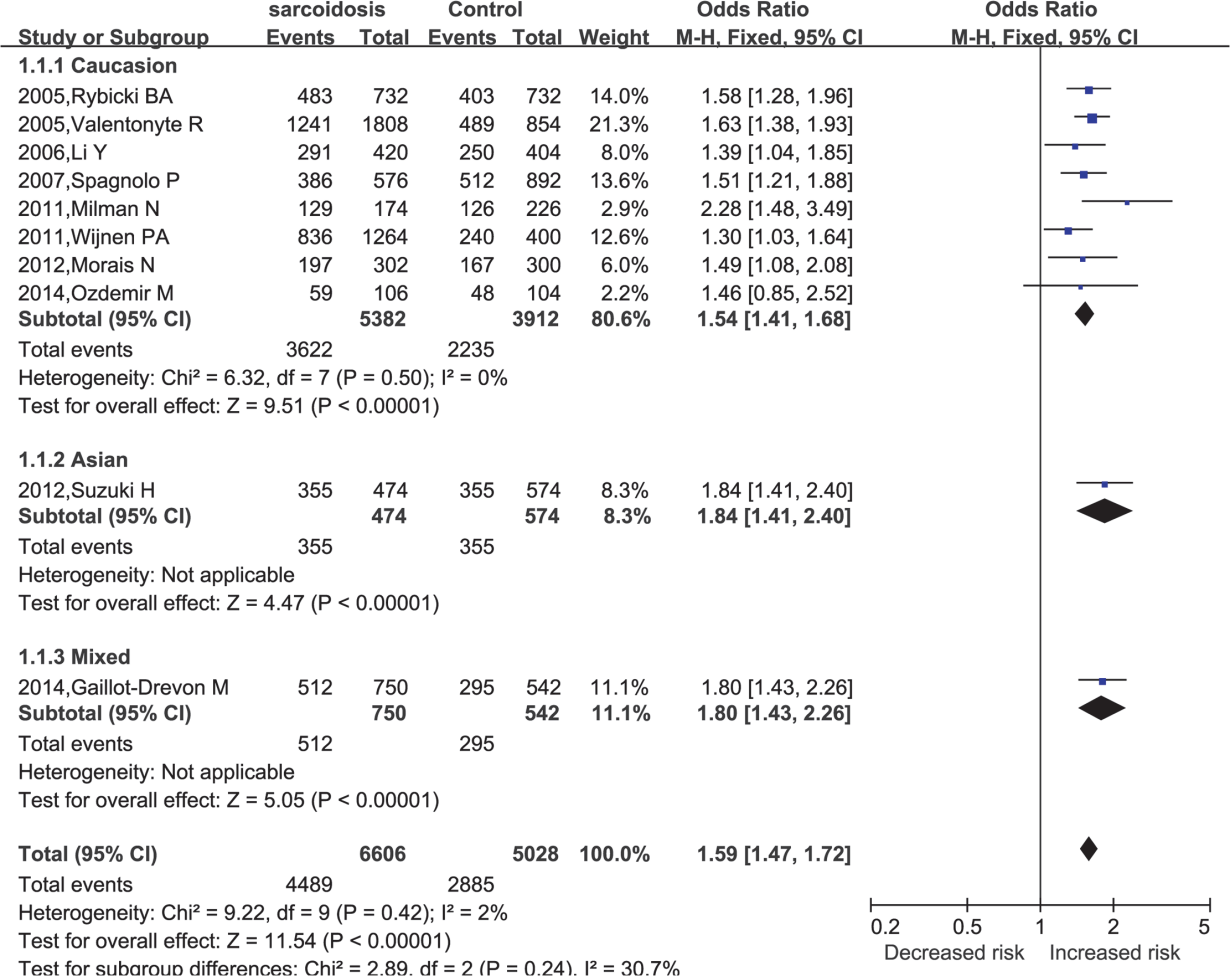
Forest plots of OR with 95% CI for the association of *BTNL2* rs2076530 polymorphism and sarcoidosis susceptibility subanalyzed by enthnicity in A *vs* G.

We found that the A allele of *BTNL2* rs2076530 was associated with an increased risk of sarcoidosis in allelic model (A *vs*. G, *P*<0.00001). The pooled OR was 1.59 (95%CI: 1.47–1.72) under the fixed effect model, without between-study heterogeneity (*I* ^2^ = 2%, *P* = 0.42) (Fig 2). In subgroup analyses by ethnicity, there were similar results in Caucasion (OR = 1.54, 95%CI: 1.41–1.68, *P*<0.00001), Asian (OR = 1.84, 95%CI: 1.41–2.40, *P*<0.00001), and mixed population (OR = 1.80, 95%CI: 1.43–2.26, *P*<0.00001) (Fig 2).

**Fig 3:**
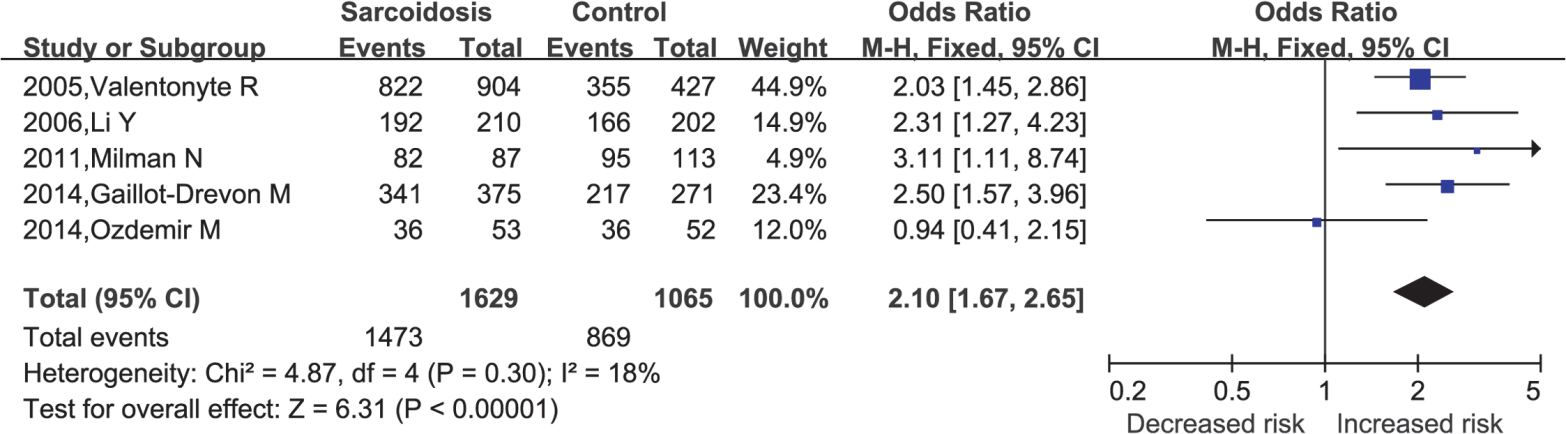
Forest plots of OR with 95% CI for the association of *BTNL2* rs2076530 polymorphism and sarcoidosis susceptibility in AA+AG *vs* GG.

**Fig 4:**
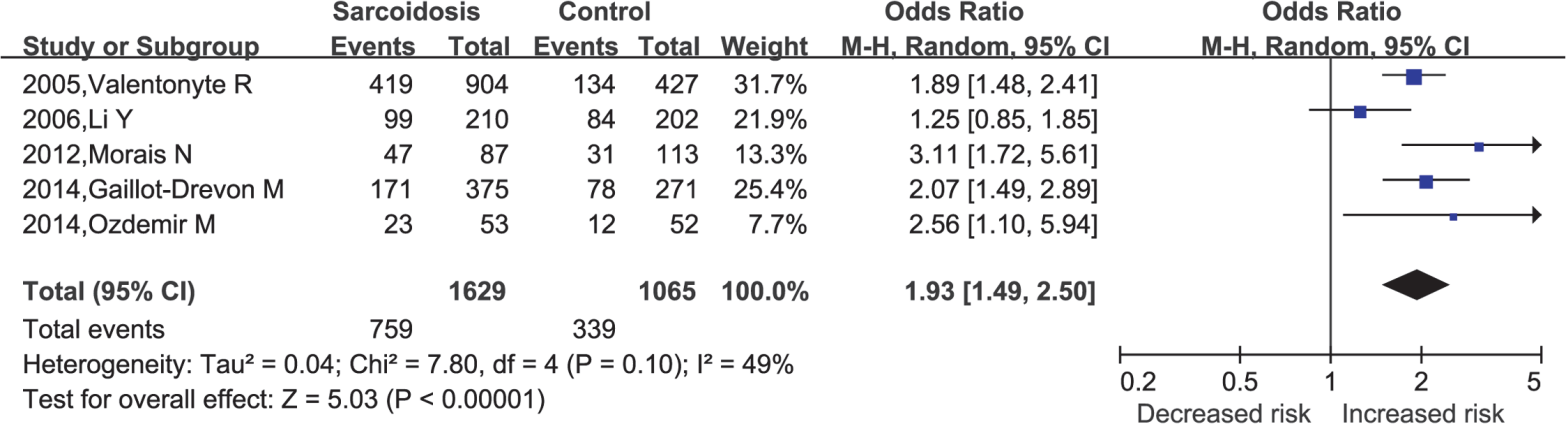
Forest plots of OR with 95% CI for the association of *BTNL2* rs2076530 polymorphism and sarcoidosis susceptibility in AA *vs* AG+GG.

Combined data from the five studies with available genotype data [4, 14,16, 19,21] showed similar results under dominant model (AA + AG *vs*. GG, OR = 2.10, 95%CI: 1.67–2.65, *P*<0.00001, Fig 3), and recessive model (AA *vs*. AG + GG, OR = 1.93, 95%CI: 1.49–2.50, *P* <0.00001, Fig 4). As most of the subjects in the five studies were Caucasion, no subgroup analyses were performed under dominant model and recessive model.

### Sensitivity analysis

We performed sensitivity analysis for statistically significant result. For the association of the *BTNL2* rs2076530 polymorphism and sarcoidosis susceptibility among the overall populations, the observed significant result was not materially altered after sequentially excluding each study.

### Publication bias

**Fig 5:**
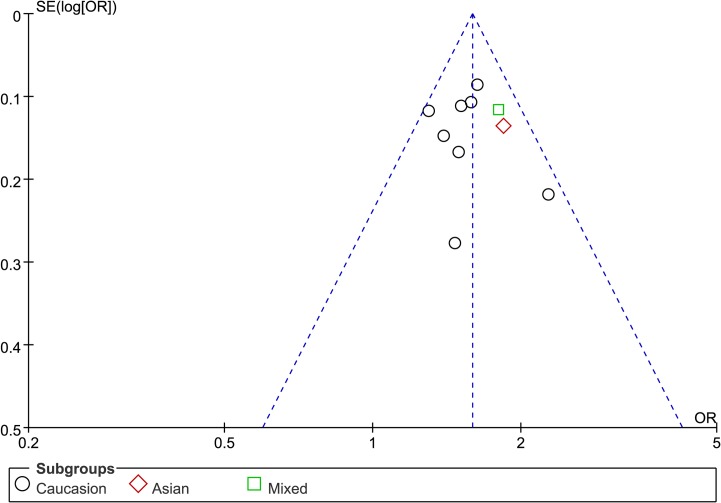
Funnel plot for evaluation of publication bias in the included studies on the association of *BTNL2* rs2076530 polymorphism and sarcoidosis susceptibility in A *vs* G.

Neither Egger’s test nor Begg’s test indicated significant publication bias (*P* = 0.956 and *P* = 0.592, respectively) in allelic model. The shape of the funnel plot was symmetrical (Fig 5).

## Discussion

Sarcoidosis has gained much attention from physicians since 1877 when it was first described. Although much progress has been made, much confusion still remains about this disease[7,8], with its cause being an improtant one.

It is assumed that genes contribute to the development of sarcoidosis since its trend of family clustering [22]and the different morbidities between ethnic groups, which have been described by several observational studies. Two previous Genome-wide association studies (GWASs) conducted in European and American populations [23,24]implicate multiple shared loci in sarcoidosis succeptibility, one of which, the single nucleotide polymorphism rs2076530 in *BTNL2* gene has been studied in many other ethnic groups. The aim of this study was to investigate the association of the *BTNL2* rs2076530 polymorphism and sarcoidosis susceptibility by conducting a meta-analysis in ten case–control studies including 5817 subjects. We provided convincing evidence on the contributory effect of *BTNL2* rs2076530 polymorphism in the development of sarcoidosis. The results of this meta-analysis are consistent with the findings of the two previous GWASs concerning sarcoidosis[23,24]. The significant increased A allele of *BTNL2* rs2076530 observed in sarcoidosis group, implys the role of this truncating single nucleotide polymorphism in sarcoidosis susceptibility.

Our study had some advantages in several key aspects. Firstly, there was no between-study heterogeneity. Secondly, no publication bias was indicated. Thirdly, there was little modification after sequentially excluding individual study. These characters of our meta-analysis indicated a reliable and stable result about the association between *BTNL2* rs2076530 polymorphism and sarcoidosis risk.

In interpreting the results of this study, the limitations of the meta-analysis should be mentioned. Firstly, sarcoidosis is a complicated and multi-factor disease and potential interactions between gene-gene and gene-enviroment should be considered. However, this meta-analysis only assessed the association of *BTNL2* rs2076530 polymorphism and sarcoidosis susceptibility. Secondly, nearly all the including studies were from the Caucasians, so the results may only be applicable to this ethnic population. In particular, it is worthy to note that *BTNL2* rs2076530 polymorphism was not strongly associated with sarcoidosis in African Americans in American GWAS [24]. Therefore further studies in other ethnic groups such as Asians, Africans and Latinos are required. Thirdly, the Asian subgroup only included one study. The small number may result in low statistical power. Fourthly, due to insufficient data reported by the original studies, further stratifications by gender, clinical course or other clinical variables were not possible.

In conclusion, this is the first meta-analysis concerning *BTNL2* rs2076530 polymorphism and sarcoidosis susceptibility up to date. This meta-analysis extended previous findings on the association between the *BTNL2* rs2076530 polymorphism and sarcoidosis, by showing that the A allele of *BTNL2* rs2076530 was associated with an increased risk of sarcoidosis susceptibility. Additional well designed large studies were needed to validate our findings. Further systematic studies should be encouraged to explore the underlying mechanisms of this association.

## Supporting Information

S1 File PRISMA Checklist. (DOC)

S2 File PRISMA Flow Diagram. (DOC)

S3 File Meta-analysis of genetic association studies checklist. (DOCX)

S4 File Details of the procedure for including eligible studies. (DOCX)
