# Supplementary material for: BTNL2 Gene Polymorphism and Sarcoidosis Susceptibility: A Meta-Analysis
Source: PLoS One. 2015 Apr 7;10(4):e0122639. doi: 10.1371/journal.pone.0122639 (PMC4388687; doi:10.1371/journal.pone.0122639)
Supplement: S1 File — (DOC) [file pone.0122639.s001.doc]

| **Section/topic** | **#** | **Checklist item** | **Reported on page #** |
| --- | --- | --- | --- |
| **TITLE** | | |  |
| Title | 1 | BTNL2 gene polymorphism and sarcoidosis susceptibility: A meta-analysis | Page 1 |
| **ABSTRACT** | | |  |
| Structured summary | 2 | Background: Butyrophilin-like 2 (BTNL2) rs2076530 gene polymorphism has been implicated in susceptibility to sarcoidosis. However, results from previous studies are conflicting. To assess the the association of BTNL2 polymorphism and sarcoidosis susceptibility, a meta-analysis was performed.  Methods: PubMed, Embase were searched for eligible case-control studies. Data were extracted and pooled odds ratios (OR) with 95% confidence intervals (CI) were calculated.  Results: Ten studies involving a total of 3303 cases and 2514 controls were included in this meta-analysis. Combined data indicated that BTNL2 rs2076530 polymorphism was associated with sarcoidosis susceptibility in allelic model (A vs. G, OR=1.59, 95%CI: 1.47-1.72), dominant model (AA + AG vs. GG, OR=2.10, 95%CI: 1.67-2.65), and recessive model (AA vs. AG + GG, OR=1.93, 95%CI: 1.49-2.50).  Conclusions: This meta-analysis indicates that BTNL2 rs2076530 polymorphism contributes to the risk of sarcoidosis. | Page 2 |
| **INTRODUCTION** | | |  |
| Rationale | 3 | Several studies have been undertaken to evaluate this potential relationship between BTNL2 rs2076530 polymorphism and sarcoidosis susceptibility. However, results from different reports of different geographic areas are not consistent. Besides, many studies of them are small in size and, as such, may on their own lack sufficient statistical power to address this issue adequately. | Page2, 3 |
| Objectives | 4 | In an effort to clarify the association of BTNL2 rs2076530 polymorphism and sarcoidosis susceptibility, we performed a meta-analysis on all the studies identified by systematic review of literatures. | Page 3 |
| **METHODS** | | |  |
| Protocol and registration | 5 | No review protocol can be accessed via the internet. No registration information or registration number can be provided. | None |
| Eligibility criteria | 6 | Reports that fulfilled the following criteria were included: (1) evaluating the BTNL2 rs2076530 polymorphism and sarcoidosis risks; (2) case-control designs on unrelated individuals; (3) odds ratios (OR) with 95% confidence intervals (CI) or sufficient data for their calculation were available; (4) the control subjects satisfied the Hardy-Weinberg equilibrium (HWE). There was no restriction on languages. | Page 3 |
| Information sources | 7 | We searched PubMed, Embase up to 4 August 2014 independently. All eligible articles were screened, and their references were checked for other relevant studies. | Page 3 |
| Search | 8 | The search strategy was as follows: (“sarcoidosis” or “Schaumann Syndrome” or “Boeck Disease” or “Besnier Boeck Schaumann Syndrome”) and (“SNP” or “variant” or “polymorphism” or “mutation”) in combination with (“BTNL2” or “butyrophilin-like 2” or “rs2076530”). | Page 3 |
| Study selection | 9 | Reports that fulfilled the following criteria were included: (1) evaluating the BTNL2 rs2076530 polymorphism and sarcoidosis risks; (2) case-control designs on unrelated individuals; (3) odds ratios (OR) with 95% confidence intervals (CI) or sufficient data for their calculation were available; (4) the control subjects satisfied the Hardy-Weinberg equilibrium (HWE). And reports were excluded if any of the following conditions existing: (1) reviews, abstracts and studies with overlapping or repeated data, (2) data about allelic frequencies could not be obtained. In case of overlapping or repeated studies, the one with most subjects was chosen. | Page 3 |
| Data collection process | 10 | The data were extracted by two investigators (Y.Lin, J.Wei) independently and a consensus was reached on all items. Any disagreement was resolved through discussion or adjudicated by a third author. | Page 4 |
| Data items | 11 | The following data was collected: first author, year, ethnicity, sample size, mean age, gender ratio, sarcoidosis criteria, source of controls, and genotype or allele distribution in cases and controls. | Page 4 |
| Risk of bias in individual studies | 12 | The data were extracted by two investigators (Y.Lin, J.Wei) independently and a consensus was reached on all items. Any disagreement was resolved through discussion or adjudicated by a third author. The following data was collected: first author, year, ethnicity, sample size, mean age, gender ratio, sarcoidosis criteria, source of controls, and genotype or allele distribution in cases and controls. | Page 4 |
| Summary measures | 13 | The strength of the associations between BTNL2 rs2076530 polymorphism and sarcoidosis risks was evaluated using OR with 95% CI. The pooled ORs were calculated for allelic model (A vs. G), dominant model (AA + AG vs. GG) and recessive model (AA vs. AG + GG) respectively. The pooled OR was calculated with a fixed-effects or random-effects model according to heterogeneity. | Page 4 |
| Synthesis of results | 14 | The data were analyzed using Review Manager 5.2 (The Nordic Cochrane Centre, The Cochrane Collaboration, Copenhagen, Denmark) and STATA 12.0 software (Stata Corp LP, College Station, TX, USA). Heterogeneity was calculated using the Q-test and I^2^-statistics. If the P-value of the Q-test was>0.10, the pooled OR was assessed in a fixed-effects model; otherwise, the random-effects model was applied. I2 values were used to quantify heterogeneity. Z-test were used to determine the significance of the pooled OR, in which a P-value of <0.05 was considered statistically significant. | Page 4 |

Page 1 of 2

| **Section/topic** | **#** | **Checklist item** | **Reported on page #** |
| --- | --- | --- | --- |
| Risk of bias across studies | 15 | Heterogeneity was calculated using the Q-test and I2-statistics. Publication bias was assessed using Egger’s test, Begg’s test and funnel plot. | Page 4 |
| Additional analyses | 16 | Subgroup analyses were performed to evaluate the ethnic-specific effects. For the subgroup analysis by ethnicity, the study populations were stratified into three groups: Caucasians, Asians, and mixed population. We also performed sensitivity analysis by extracting a single study each time to check the stability of the results. | Page 4 |
| **RESULTS** | | |  |
| Study selection | 17 | A total of 84 articles were retrieved after initial search. Among them, twenty-five duplicate records were removed. After evaluating titles and abstracts, forty-seven results were excluded for not relating to original study about association of BTNL2 rs2076530 polymorphism and sarcoidosis susceptibility. After reading the full text, two articles were excluded for no enough data available. Finally, ten articles were included. The procedure for including eligible studies is showed in Figure 1 | Page 5 |
| Study characteristics | 18 | Ten articles were included (the subjects of eight studies were Caucasion population, one study included Asian population and one study involved mixed population), containing 3303 cases and 2514 controls. The general characteristics of all the including studies are listed in Table 1. | Page 5 |
| Risk of bias within studies | 19 | See table 1 | Page 9 |
| Results of individual studies | 20 | See figure 2,3,4. | Page 5 |
| Synthesis of results | 21 | We found that the A allele of BTNL2 rs2076530 was associated with an increased risk of sarcoidosis in allelic model (A vs. G, P<0.00001). The pooled OR was 1.59 (95%CI: 1.47-1.72) under the fixed effect model, without between-study heterogeneity (I^2^ =2%, P =0.42) (Figure 2). Combined data from the five studies showed similar results under dominant model (AA + AG vs. GG, OR=2.10, 95%CI: 1.67-2.65, P<0.00001, Figure 3), and recessive model (AA vs. AG + GG, OR=1.93, 95%CI: 1.49-2.50, P <0.00001, Figure 4). | Page 5 |
| Risk of bias across studies | 22 | Neither Egger’s test nor Begg’s test indicated significant publication bias (P = 0.956 and P = 0.592, respectively) in allelic model. The shape of the funnel plot was symmetrical (Figure 5). | Page 6 |
| Additional analysis | 23 | In subgroup analyses by ethnicity, there were similar results in Caucasion (OR=1.54, 95%CI: 1.41-1.68, P<0.00001), Asian (OR=1.84, 95%CI: 1.41-2.40, P<0.00001), and mixed population (OR=1.80, 95%CI: 1.43-2.26, P<0.00001) (Figure 2).We performed sensitivity analysis for statistically significant result. For the association of the BTNL2 rs2076530 polymorphism and sarcoidosis susceptibility among the overall populations, the observed significant result was not materially altered after sequentially excluding each study. | Page5, 6 |
| **DISCUSSION** | | |  |
| Summary of evidence | 24 | Combined data indicated that BTNL2 rs2076530 polymorphism was associated with sarcoidosis susceptibility in allelic model (A vs. G, OR=1.59, 95%CI: 1.47-1.72), dominant model (AA + AG vs. GG, OR=2.10, 95%CI: 1.67-2.65), and recessive model. | Page 5 |
| Limitations | 25 | Firstly, sarcoidosis is a complicated and multi-factor disease and potential interactions between gene-gene and gene-enviroment should be considered. However, this meta-analysis only assessed the the association of BTNL2 rs2076530 polymorphism and sarcoidosis susceptibility. Secondly, nearly all the including studies were from the Caucasians, so the results may only be applicable to this ethnic population. Further studies in other ethnic groups such as Asians, Africans and Latinos are required. Thirdly, the Asian subgroup only included one study. The small number may result in low statistical power. Fourthly, due to insufficient data reported by the original studies, further stratifications by gender, clinical course or other clinical variables were not possible. | Page 7 |
| Conclusions | 26 | This meta-analysis extended previous findings on the association between the BTNL2 rs2076530 polymorphism and sarcoidosis, by showing that the A allele of BTNL2 rs2076530 was associated with an increased risk of sarcoidosis susceptibility. | Page 7 |
| **FUNDING** | | |  |
| Funding | 27 | There is no funding for this systematic review. | None |

*From:*  Moher D, Liberati A, Tetzlaff J, Altman DG, The PRISMA Group (2009). Preferred Reporting Items for Systematic Reviews and Meta-Analyses: The PRISMA Statement. PLoS Med 6(6): e1000097. doi:10.1371/journal.pmed1000097

For more information, visit: **www.prisma-statement.org**.

Page 2 of 2
