# Supplementary material for: BTNL2 Gene Polymorphism and Sarcoidosis Susceptibility: A Meta-Analysis
Source: PLoS One. 2015 Apr 7;10(4):e0122639. doi: 10.1371/journal.pone.0122639 (PMC4388687; doi:10.1371/journal.pone.0122639)
Supplement: S2 File — (DOC) [file pone.0122639.s002.doc]

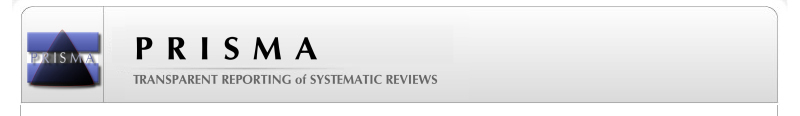
**PRISMA 2009 Flow Diagram**

**Screening**

**Included**

**Eligibility**

**Identification**

Records identified through database searching
(n = 84 )

Additional records identified through other sources
(n = 0 )

Records after duplicates removed
(n = 59 )

Records screened
(n = 59 )

Records excluded
(n = 47 )

Full-text articles assessed for eligibility
(n = 12 )

Full-text articles excluded, with reasons
(n = 2 )

Studies included in qualitative synthesis
(n = 10 )

Studies included in quantitative synthesis (meta-analysis)
(n = 10 )
