# Supplementary material for: BTNL2 Gene Polymorphism and Sarcoidosis Susceptibility: A Meta-Analysis
Source: PLoS One. 2015 Apr 7;10(4):e0122639. doi: 10.1371/journal.pone.0122639 (PMC4388687; doi:10.1371/journal.pone.0122639)
Supplement: S4 File — (DOCX) [file pone.0122639.s004.docx]

**Potential relevant articles identified through Pubmed: n=32**

1. Sarcoidosis Vasc Diffuse Lung Dis. 2014 Jul 8;31(2):136-41.

Lack of correlation of BTNL2 polymorphism and cancer risk in sarcoidosis. BTNL2 and cancer risk in sarcoidosis.

Gaillot-Drevon M(1), Calender A, Blay JY, Valeyre D, Israel-Biet D, Roy P,Pacheco Y.

2. BMC Genet. 2013 Jul 6;14:61. doi: 10.1186/1471-2156-14-61.

A common 56-kilobase deletion in a primate-specific segmental duplication creates a novel butyrophilin-like protein.

Aigner J(1), Villatoro S, Rabionet R, Roquer J, Jiménez-Conde J, Martí E, Estivill X.

3. Hum Genet. 2013 Jul;132(7):803-10. doi: 10.1007/s00439-013-1292-5. Epub 2013 Mar 31.

Replication of genetic loci for sarcoidosis in US black women: data from the Black Women's Health Study.

Cozier Y(1), Ruiz-Narvaez E, McKinnon C, Berman J, Rosenberg L, Palmer J.

4. Respir Med. 2012 Dec;106(12):1771-7. doi: 10.1016/j.rmed.2012.08.009. Epub 2012 Sep 25.

BTNL2 gene polymorphism associations with susceptibility and phenotype expression in sarcoidosis.

Morais A(1), Lima B, Peixoto MJ, Alves H, Marques A, Delgado L.

5. Invest Ophthalmol Vis Sci. 2012 Oct 11;53(11):7109-15. doi:10.1167/iovs.12-10491.

Genetic characterization and susceptibility for sarcoidosis in Japanese patients: risk factors of BTNL2 gene polymorphisms and HLA class II alleles.

Suzuki H(1), Ota M, Meguro A, Katsuyama Y, Kawagoe T, Ishihara M, Asukata Y,

Takeuchi M, Ito N, Shibuya E, Nomura E, Uemoto R, Nishide T, Namba K, Kitaichi N,

Morimoto S, Kaburaki T, Ando Y, Takenaka S, Nakamura J, Saeki K, Ohno S, Inoko H,

Mizuki N.

6. Rev Mal Respir. 2011 Apr;28(4):409-18. doi: 10.1016/j.rmr.2010.09.035. Epub 2011 Mar 16.

[Sarcoidosis and genetics].

Pacheco Y.

7. Clin Respir J. 2011 Apr;5(2):105-11. doi: 10.1111/j.1752-699X.2010.00206.x.

The BTNL2 A allele variant is frequent in Danish patients with sarcoidosis.

Milman N(1), Svendsen CB, Nielsen FC, van Overeem Hansen T.

8. Hum Immunol. 2011 Apr;72(4):342-7. doi: 10.1016/j.humimm.2011.01.011. Epub 2011 Jan 20.

Butyrophilin-like 2 in pulmonary sarcoidosis: a factor for susceptibility and progression?

Wijnen PA(1), Voorter CE, Nelemans PJ, Verschakelen JA, Bekers O, Drent M.

9. Rev Med Interne. 2011 Feb;32(2):73-9. doi: 10.1016/j.revmed.2010.09.011. Epub 2010 Nov 9.

[Pathogenesis of sarcoidosis].

Pacheco Y.

10. Sarcoidosis Vasc Diffuse Lung Dis. 2009 Jul;26(2):162-6.

Homozygous variant rs2076530 of BTNL2 and familial sarcoidosis.

Coudurier M(1), Freymond N, Aissaoui S, Calender A, Pacheco Y, Devouassoux G.

11. Infect Genet Evol. 2010 May;10(4):517-21. doi: 10.1016/j.meegid.2010.02.006. Epub 2010 Feb 20.

Analysis of the association between BTNL2 polymorphism and tuberculosis in Chinese Han population.

Lian Y(1), Yue J, Han M, Liu J, Liu L.

12. Pneumologie. 2009 Mar;63(3):166-75.

[Genetics of sarcoidosis: a key to understanding its pathogenesis].

Müller-Quernheim J(1), Schürmann M, Hofmann S, Gaede KI, Fischer A, Prasse A,

Zissel G, Schreiber S.

13. Tissue Antigens. 2009 Jan;73(1):59-61. doi: 10.1111/j.1399-0039.2008.01180.x.

Extended genetic analysis of BTNL2 in sarcoidosis.

Li Y(1), Pabst S, Lokhande S, Grohé C, Wollnik B.

14. Allergol Int. 2009 Mar;58(1):29-35. doi: 10.2332/allergolint.08-OA-0005. Epub 2008 Dec 1.

Genetic impact of a butyrophilin-like 2 (BTNL2) gene variation on specific IgE responsiveness to Dermatophagoides farinae (Der f) in Japanese.

Konno S(1), Takahashi D, Hizawa N, Hattori T, Takahashi A, Isada A, Maeda Y, Huang SK, Nishimura M.

15. Curr Opin Pulm Med. 2008 Sep;14(5):434-9. doi: 10.1097/MCP.0b013e3283043de7.

Genetics of sarcoidosis.

Grunewald J.

16. Respiration. 2007;74(6):601-8.

Genetic predisposition to respiratory diseases: infiltrative lung diseases.

Steele MP(1), Brown KK.

17. Cardiology. 2008;109(2):117-21. Epub 2007 Aug 17.

Isolated cardiac sarcoidosis associated with the expression of a splice variant coding for a truncated BTNL2 protein.

Meyer T(1), Lauschke J, Ruppert V, Richter A, Pankuweit S, Maisch B.

18. Proc Am Thorac Soc. 2007 Aug 15;4(5):457-60.

Advances in the genetics of sarcoidosis.

Iannuzzi MC.

19. Tissue Antigens. 2007 Sep;70(3):219-27.

Analysis of BTNL2 genetic polymorphisms in British and Dutch patients with sarcoidosis.

Spagnolo P(1), Sato H, Grutters JC, Renzoni EA, Marshall SE, Ruven HJ, Wells AU,

Tzouvelekis A, van Moorsel CH, van den Bosch JM, du Bois RM, Welsh KI.

20. Tissue Antigens. 2007 Aug;70(2):128-35.

Butyrophilin-like 2 gene is associated with ulcerative colitis in the Japanese under strong linkage disequilibrium with HLA-DRB1*1502.

Mochida A(1), Kinouchi Y, Negoro K, Takahashi S, Takagi S, Nomura E, Kakuta Y,

Tosa M, Shimosegawa T.

21. Clin Dermatol. 2007 May-Jun;25(3):242-9.

Genetics of sarcoidosis.

Spagnolo P(1), du Bois RM.

22. Tissue Antigens. 2007 Mar;69(3):236-41.

Analysis of the BTNL2 truncating splice site mutation in tuberculosis, leprosy and Crohn's disease.

Johnson CM(1), Traherne JA, Jamieson SE, Tremelling M, Bingham S, Parkes M, Blackwell JM, Trowsdale J.

23. Microbes Infect. 2007 Apr;9(4):522-8. Epub 2007 Jan 27.

Allelic variation in BTNL2 and susceptibility to tuberculosis in a South African population.

Möller M(1), Kwiatkowski R, Nebel A, van Helden PD, Hoal EG, Schreiber S.

24. Semin Respir Crit Care Med. 2007 Feb;28(1):15-21.

Genetics of sarcoidosis.

Iannuzzi MC.

25. Am J Respir Crit Care Med. 2007 Mar 1;175(5):498-506. Epub 2006 Dec 14.

Expression of receptor for advanced glycation end products in sarcoid granulomas.

Campo I(1), Morbini P, Zorzetto M, Tinelli C, Brunetta E, Villa C, Bombieri C,Cuccia M, Agostini C, Bozzi V, Facoetti A, Ferrarotti I, Mazzola P, Scabini R,Semenzato G, Pignatti PF, Pozzi E, Luisetti M.

26. Clin Endocrinol (Oxf). 2006 Oct;65(4):429-32.

Association of the BTNL2 rs2076530 single nucleotide polymorphism with Graves' disease appears to be secondary to DRB1 exon 2 position beta74.

Simmonds MJ(1), Heward JM, Barrett JC, Franklyn JA, Gough SC.

27. J Immunol. 2006 Jun 15;176(12):7354-60.

BTNL2, a butyrophilin-like molecule that functions to inhibit T cell activation.

Nguyen T(1), Liu XK, Zhang Y, Dong C.

28. BMC Med Genet. 2006 Mar 9;7:21.

On the Wegener granulomatosis associated region on chromosome 6p21.3.

Szyld P(1), Jagiello P, Csernok E, Gross WL, Epplen JT.

29. Thorax. 2006 Mar;61(3):273-4.

BTNL2 gene variant and sarcoidosis.

Li Y, Wollnik B, Pabst S, Lennarz M, Rohmann E, Gillissen A, Vetter H, Grohé C.

30. Hum Mol Genet. 2006 Jan 1;15(1):155-61. Epub 2005 Dec 1.

Association of the truncating splice site mutation in BTNL2 with multiple sclerosis is secondary to HLA-DRB1*15.

Traherne JA(1), Barcellos LF, Sawcer SJ, Compston A, Ramsay PP, Hauser SL, Oksenberg JR, Trowsdale J.

31. Am J Hum Genet. 2005 Sep;77(3):491-9. Epub 2005 Jul 20.

The BTNL2 gene and sarcoidosis susceptibility in African Americans and Whites.

Rybicki BA(1), Walewski JL, Maliarik MJ, Kian H, Iannuzzi MC; ACCESS Research Group.

32. Nat Genet. 2005 Apr;37(4):357-64. Epub 2005 Feb 27.

Sarcoidosis is associated with a truncating splice site mutation in BTNL2.

Valentonyte R(1), Hampe J, Huse K, Rosenstiel P, Albrecht M, Stenzel A, Nagy M, Gaede KI, Franke A, Haesler R, Koch A, Lengauer T, Seegert D, Reiling N, Ehlers S, Schwinger E, Platzer M, Krawczak M, Müller-Quernheim J, Schürmann M, Schreiber S.

**Potential relevant articles identified through Embase: n=52**

1.

Sarcoidosis and uveitis.

Jamilloux Y., Kodjikian L., Broussolle C., Seve P.

Autoimmunity Reviews. 13 (8) (pp 840-849), 2014. Date of Publication: August 2014.

2.

Is there a genetic predisposition for Turkish patients with sarcoidosis in the 329-bp region containing the BTNL2 rs2076530 polymorphism?.

Ozdemir M., Saydam F., Kurt E., Degirmenci I., Tuncel T., Cilingir O., Gunes H.V., Artan S.

Turkish Journal of Medical Sciences. 44 (4) (pp 590-594), 2014. Date of Publication: 2014.

3.

Major histocompatibility complex class II and BTNL2 associations in sarcoidosis.

Wennerstrom A., Pietinalho A., Lasota J., Salli K., Surakka I., Seppanen M., Selroos O., Lokki M.-L.

European Respiratory Journal. 42 (2) (pp 550-553), 2013. Date of Publication: 01 Aug 2013.

4.

Characterization of the lung microbiome in sarcoidosis and healthy individuals with respect to host genotype.

Zimmermann A., Hasler R., Zissel G., Gaede K.I., Muller-Quernheim J., Prasse A., Fischer A., Nebel A., Schreiber S.

Pneumologie. Conference: Chronic Inflammatory Disorders of the Lung 2012 Freiburg Germany. Conference Start: 20120928 Conference End: 20120929. Conference Publication: (var.pagings). 66 (11) , 2012. Date of Publication: November 2012.

5.

A common 56-kilobase deletion in a primate-specific segmental duplication creates a novel butyrophilin-like protein.

Aigner J., Villatoro S., Rabionet R., Roquer J., Jimenez-Conde J., Marti E., Estivill X.

BMC Genetics. 14 , 2013. Article Number: 61. Date of Publication: 06 Jul 2013.

6.

Novel MHC associations in sarcoidosis.

Wennerstrom A., Pietinalho A., Salli K., Lasota J., Selroos O., Lokki M.-L.

Tissue Antigens. Conference: 27th EFI European Immunogenetics and Histocompatibility Conference Maastricht Netherlands. Conference Start: 20130511 Conference End: 20130514. Conference Publication: (var.pagings). 81 (5) (pp 375), 2013. Date of Publication: May 2013.

7.

Selected non-MHC variants and acute graft vs host disease after allogeneic haematopoietic stem cell transplantation.

Mrazek F., Kriegova E., Ambruzova Z., Staffova K., Stahelova A., Raida L., Indrak K., Petrek M.

Tissue Antigens. Conference: 27th EFI European Immunogenetics and Histocompatibility Conference Maastricht Netherlands. Conference Start: 20130511 Conference End: 20130514. Conference Publication: (var.pagings). 81 (5) (pp 317-318), 2013. Date of Publication: May 2013.

8.

Genome-wide association study of African and European Americans implicates multiple shared and ethnic specific loci in sarcoidosis susceptibility.

Adrianto I., Lin C.P., Hale J.J., Levin A.M., Datta I., Parker R., Adler A., Kelly J.A., Kaufman K.M., Lessard C.J., Moser K.L., Kimberly R.P., Harley J.B., Iannuzzi M.C., Rybicki B.A., Montgomery C.G.

PloS one. 7 (8) , 2012. Article Number: e43907. Date of Publication: 2012.

9.

Genetic characterization and susceptibility for sarcoidosis in Japanese patients: risk factors of BTNL2 gene polymorphisms and HLA class II alleles.

Suzuki H., Ota M., Meguro A., Katsuyama Y., Kawagoe T., Ishihara M., Asukata Y., Takeuchi M., Ito N., Shibuya E., Nomura E., Uemoto R., Nishide T., Namba K., Kitaichi N., Morimoto S., Kaburaki T., Ando Y., Takenaka S., Nakamura J., Saeki K., Ohno S., Inoko H., Mizuki N.

Investigative ophthalmology & visual science. 53 (11) (pp 7109-7115), 2012. Date of Publication: Oct 2012.

10.

Replication of genetic loci for sarcoidosis in US black women: Data from the Black Women's Health Study.

Cozier Y., Ruiz-Narvaez E., McKinnon C., Berman J., Rosenberg L., Palmer J.

Human Genetics. 132 (7) (pp 803-810), 2013. Date of Publication: July 2013.

11.

Evaluation of BTNL2 gene mutation in rs2076530 allele in patients with the diagnosis of skin sarcoidosis.

Hesari K.K., Montaser-Kouhsari L., Taleb S., Hashemi M.A., Rahimpour S., Firooz A.

American Journal of Dermatopathology. Conference: 31st Symposium of the ISDP Barcelona Spain. Conference Start: 20101021 Conference End: 20101023. Conference Publication: (var.pagings). 33 (2) (pp 208), 2011. Date of Publication: April 2011.

12.

On the Wegener granulomatosis associated region on chromosome 6p21.3.

Szyld P., Jagiello P., Csernok E., Gross W.L., Epplen J.T.

BMC medical genetics. 7 , 2006. Article Number: 21. Date of Publication: 2006.

13.

Genetic predisposition to cutaneous sarcoidosis.

Shahkarami S., Rezaei N.

Acta Medica Iranica. 50 (10) (pp 721-722), 2012. Date of Publication: 2012.

14.

BTNL2 gene polymorphism associations with susceptibility and phenotype expression in sarcoidosis.

Morais A., Lima B., Peixoto M.J., Alves H., Marques A., Delgado L.

Respiratory Medicine. 106 (12) (pp 1771-1777), 2012. Date of Publication: December 2012.

15.

Genome-wide association study of African and European Americans implicates multiple shared and ethnic specific loci in sarcoidosis susceptibility.

Adrianto I., Lin C.P., Hale J.J., Levin A.M., Datta I., Parker R., Adler A., Kelly J.A., Kaufman K.M., Lessard C.J., Moser K.L., Kimberly R.P., Harley J.B., Iannuzzi M.C., Rybicki B.A., Montgomery C.G.

PLoS ONE. 7 (8) , 2012. Article Number: e43907. Date of Publication: 27 Aug 2012.

16.

A genome-wide association study identifies new candidate loci associated with sarcoidosis in european americans.

Adrianto I., Lin C.P., Lessard C.J., Datta I., Levin A.M., Parker R., Adler A., Kaufman K.M., Moser K.L., Iannuzzi M.C., Rybicki B.A., Montgomery C.G.

American Journal of Respiratory and Critical Care Medicine. Conference: American Thoracic Society International Conference, ATS 2011 Denver, CO United States. Conference Start: 20110513 Conference End: 20110518. Conference Publication: (var.pagings). 183 (1 MeetingAbstracts) , 2011. Date of Publication: 01 May 2011.

17.

Genome-wide association study of African Americans implicates multiple lung and inflammatory disease-associated loci in sarcoidosis susceptibility.

Adrianto I., Lin C.P., Hale J.J., Levin A.M., Datta I., Parker R., Adler A.

Arthritis and Rheumatism. Conference: Annual Scientific Meeting of the American College of Rheumatology and Association of Rheumatology Health Professionals 2011 Chicago, IL United States. Conference Start: 20111104 Conference End: 20111109. Conference Publication: (var.pagings). 63 (10 SUPPL. 1) , 2011. Date of Publication: October 2011.

18.

Butyrophilin-like 2 and HLA-DRB1*15: Markers for susceptibility and progression in pulmonary sarcoidosis.

Voorter C.E.M., Wijnen P.A., Nelemans P.J., Verschakelen J.A., Bekers O., Drent M.

Tissue Antigens. Conference: 25th European Immunogenetics and Histocompatibility Conference Prague Czech Republic. Conference Start: 20110504 Conference End: 20110507. Conference Publication: (var.pagings). 77 (5) (pp 395), 2011. Date of Publication: May 2011.

19.

Sarcoidosis and genetics. <Sarcoidose et genetique.>

Pacheco Y.

Revue des Maladies Respiratoires. 28 (4) (pp 409-418), 2011. Date of Publication: April 2011.

20.

The BTNL2 A allele variant is frequent in Danish patients with sarcoidosis.

Milman N., Svendsen C.B., Nielsen F.C., van Overeem Hansen T.

Clinical Respiratory Journal. 5 (2) (pp 105-111), 2011. Date of Publication: April 2011.

21.

Butyrophilin-like 2 in pulmonary sarcoidosis: A factor for susceptibility and progression?.

Wijnen P.A., Voorter C.E., Nelemans P.J., Verschakelen J.A., Bekers O., Drent M.

Human Immunology. 72 (4) (pp 342-347), 2011. Date of Publication: April 2011.

22.

Pathogenesis of sarcoidosis. <Ethiopathogenie de la sarcoidose.>

Pacheco Y.

Revue de Medecine Interne. 32 (2) (pp 73-79), 2011. Date of Publication: February 2011.

23.

Rheumatic manifestations of systemic disease: Sarcoidosis.

Chatham W.

Current Opinion in Rheumatology. 22 (1) (pp 85-90), 2010. Date of Publication: January 2010.

24.

Analysis of the association between BTNL2 polymorphism and tuberculosis in Chinese Han population.

Lian Y., Yue J., Han M., Liu J., Liu L.

Infection, Genetics and Evolution. 10 (4) (pp 517-521), 2010. Date of Publication: May 2010.

25.

Homozygous variant rs2076530 of BTNL2 and familial sarcoidosis.

Coudurier M., Freymond N., Aissaoui S., Calender A., Pacheco Y., Devouassoux G.

Sarcoidosis Vasculitis and Diffuse Lung Diseases. 26 (2) (pp 162-166), 2009. Date of Publication: 2009.

26.

Genetic impact of a butyrophilin-like 2 (BTNL2) gene variation on specific IgE responsiveness to dermatophagoides farinae (Der f) in Japanese.

Konno S., Takahashi D., Hizawa N., Hattori T., Takahashi A., Isada A., Maeda Y., Huang S.-K., Nishimura M.

Allergology International. 58 (1) (pp 29-35), 2009. Date of Publication: 2009.

27.

Extended genetic analysis of BTNL2 in sarcoidosis.

Li Y., Pabst S., Lokhande S., Grohe C., Wollnik B.

Tissue Antigens. 73 (1) (pp 59-61), 2009. Date of Publication: January 2009.

28.

BTNL2 polymorphism and cardiac sarcoidosis.

Meyer T., Lauschke J., Ruppert V., Richter A., Pankuweit S., Maisch B.

Cardiology. 112 (1) (pp 78-79), 2009. Date of Publication: November 2008.

29.

Cardiac sarcoidosis associated with BTNL2.

Becker C.D., Sridhar P., Iannuzzi M.C.

Cardiology. 112 (1) (pp 76-77), 2009. Date of Publication: November 2008.

30.

Genetics of sarcoidosis.

Grunewald J.

Current Opinion in Pulmonary Medicine. 14 (5) (pp 434-439), 2008. Date of Publication: September 2008.

31.

Prediction of disease-associated single nucleotide polymorphisms using virtual genomes constructed from a public haplotype database.

Toyabe S.-I., Miyashita A., Kitamura N., Kuwano R., Akazawa K.

Methods of Information in Medicine. 47 (6) (pp 522-528), 2008. Date of Publication: 2008.

32.

Cosignaling complexity gets more convoluted: The emerging importance of the B7-like butyrophilin family of immune regulators.

Arnett H.A., Swanson R.M., Viney J.L.

Current Immunology Reviews. 4 (1) (pp 43-52), 2008. Date of Publication: February 2008.

33.

Isolated cardiac sarcoidosis associated with the expression of a splice variant coding for a truncated BTNL2 protein.

Meyer T., Lauschke J., Ruppert V., Richter A., Pankuweit S., Maisch B.

Cardiology. 109 (2) (pp 117-121), 2008. Date of Publication: January 2008.

34.

Genetic predisposition to respiratory diseases: Infiltrative lung diseases.

Steele M.P., Brown K.K.

Respiration. 74 (6) (pp 601-608), 2007. Date of Publication: November 2007.

35.

BTNL2 allele associations with chronic beryllium disease in HLA-DPB1*Glu69-negative individuals.

Sato H., Spagnolo P., Silveira L., Welsh K.I., Du Bois R.M., Newman L.S., Maier L.A.

Tissue Antigens. 70 (6) (pp 480-486), 2007. Date of Publication: December 2007.

36.

Advances in the genetics of sarcoidosis.

Iannuzzi M.C.

Proceedings of the American Thoracic Society. 4 (5) (pp 457-460), 2007. Date of Publication: August 2007.

37.

Analysis of BTNL2 genetic polymorphisms in British and Dutch patients with sarcoidosis.

Spagnolo P., Sato H., Grutters J.C., Renzoni E.A., Marshall S.E., Ruven H.J.T., Wells A.U., Tzouvelekis A., Van Moorsel C.H.M., Van Den Bosch J.M.M., Du Bois R.M., Welsh K.I.

Tissue Antigens. 70 (3) (pp 219-227), 2007. Date of Publication: September 2007.

38.

Butyrophilin-like 2 gene is associated with ulcerative colitis in the Japanese under strong linkage disequilibrium with HLA-DRB1*1502.

Mochida A., Kinouchi Y., Negoro K., Takahashi S., Takagi S., Nomura E., Kakuta Y., Tosa M., Shimosegawa T.

Tissue Antigens. 70 (2) (pp 128-135), 2007. Date of Publication: August 2007.

39.

Genetics of sarcoidosis.

Spagnolo P., du Bois R.M.

Clinics in Dermatology. 25 (3) (pp 242-249), 2007. Date of Publication: May/June 2007.

40.

Analysis of the BTNL2 truncating splice site mutation in tuberculosis, leprosy and Crohn's disease.

Johnson C.M., Traherne J.A., Jamieson S.E., Tremelling M., Bingham S., Parkes M., Blackwell J.M., Trowsdale J.

Tissue Antigens. 69 (3) (pp 236-241), 2007. Date of Publication: March 2007.

41.

Allelic variation in BTNL2 and susceptibility to tuberculosis in a South African population.

Moller M., Kwiatkowski R., Nebel A., van Helden P.D., Hoal E.G., Schreiber S.

Microbes and Infection. 9 (4) (pp 522-528), 2007. Date of Publication: April 2007.

42.

Genetics of sarcoidosis.

Iannuzzi M.C.

Seminars in Respiratory and Critical Care Medicine. 28 (1) (pp 15-21), 2007. Date of Publication: February 2007.

43.

Expression of receptor for advanced glycation end products in sarcoid granulomas.

Campo I., Morbini P., Zorzetto M., Tinelli C., Brunetta E., Villa C., Bombieri C., Cuccia M., Agostini C., Bozzi V., Facoetti A., Ferrarotti I., Mazzola P., Scabini R., Semenzato G., Pignatti P.F., Pozzi E., Luisetti M.

American Journal of Respiratory and Critical Care Medicine. 175 (5) (pp 498-506), 2007. Date of Publication: 01 Mar 2007.

44.

BTNL2, a butyrophilin/B7-like molecule, is a negative costimulatory molecule modulated in intestinal inflammation.

Arnett H.A., Escobar S.S., Gonzalez-Suarez E., Budelsky A.L., Steffen L.A., Boiani N., Zhang M., Siu G., Brewer A.W., Viney J.L.

Journal of Immunology. 178 (3) (pp 1523-1533), 2007. Date of Publication: 01 Feb 2007.

45.

Association of the BTNL2 rs2076530 single nucleotide polymorphism with Graves' disease appears to be secondary to DRB1 exon 2 position beta74.

Simmonds M.J., Heward J.M., Barrett J.C., Franklyn J.A., Gough S.C.L.

Clinical Endocrinology. 65 (4) (pp 429-432), 2006. Date of Publication: October 2006.

46.

BTNL2, a butyrophilin-like molecule that functions to inhibit T cell activation.

Nguyen T., Liu X.K., Zhang Y., Dong C.

Journal of Immunology. 176 (12) (pp 7354-7360), 2006. Date of Publication: 15 Jun 2006.

47.

On the Wegener granulomatosis associated region on chromosome 6p21.3.

Szyld P., Jagiello P., Csernok E., Gross W.L., Epplen J.T.

BMC Medical Genetics. 7 , 2006. Article Number: 21. Date of Publication: 09 Mar 2006.

48.

BTNL2 gene variant and sarcoidosis [4].

Li Y., Wollnik B., Pabst S., Lennarz M., Rohmann E., Gillissen A., Vetter H., Grohe C.

Thorax. 61 (3) (pp 273-274), 2006. Date of Publication: March 2006.

49.

Association of the truncating splice site mutation in BTNL2 with multiple sclerosis is secondary to HLA-DRB1*15.

Traherne J.A., Barcellos L.F., Sawcer S.J., Compston A., Ramsay P.P., Hauser S.L., Oksenberg J.R., Trowsdale J.

Human Molecular Genetics. 15 (1) (pp 155-161), 2006. Date of Publication: 01 Jan 2006.

50.

The BTNL2 gene and sarcoidosis susceptibility in African Americans and whites.

Rybicki B.A., Walewski J.L., Maliarik M.J., Kian H., Iannuzzi M.C.

American Journal of Human Genetics. 77 (3) (pp 491-499), 2005. Date of Publication: September 2005.

51.

Erratum: Sarcoidosis is associated with a truncating splice site mutation in the gene BTNL2 (Nature Genetics (2005) 37 (357-364)).

Valentonyte R., Hampe J., Huse K., Rosenstiel P., Albrecht M., Stenzel A., Nagy M., Gaede K.I., Franke A., Haesler R., Koch A., Lengauer T., Seegert D., Reiling N., Ehlers S., Schwinger E., Platzer M., Krawczak M., Muller-Quernheim J., Schurmann M., Schreiber S.

Nature Genetics. 37 (6) (pp 652), 2005. Date of Publication: June 2005.

52.

Sarcoidosis is associated with a truncating splice site mutation in BTNL2.

Valentonyte R., Hampe J., Huse K., Rosenstiel P., Albrecht M., Stenzel A., Nagy M., Gaede K.I., Franke A., Haesler R., Koch A., Lengauer T., Seegert D., Reiling N., Ehlers S., Schwinger E., Platzer M., Krawczak M., Muller-Quernheim J., Schurmann M., Schreiber S.

Nature Genetics. 37 (4) (pp 357-364), 2005. Date of Publication: April 2005.

**Duplicate records: n=32**

1.

BMC Genet. 2013 Jul 6;14:61. doi: 10.1186/1471-2156-14-61.

A common 56-kilobase deletion in a primate-specific segmental duplication creates a novel butyrophilin-like protein.

Aigner J(1), Villatoro S, Rabionet R, Roquer J, Jiménez-Conde J, Martí E, Estivill X.

2.

Hum Genet. 2013 Jul;132(7):803-10. doi: 10.1007/s00439-013-1292-5. Epub 2013 Mar 31.

Replication of genetic loci for sarcoidosis in US black women: data from the Black Women's Health Study.

Cozier Y(1), Ruiz-Narvaez E, McKinnon C, Berman J, Rosenberg L, Palmer J.

3.

Respir Med. 2012 Dec;106(12):1771-7. doi: 10.1016/j.rmed.2012.08.009. Epub 2012 Sep 25.

BTNL2 gene polymorphism associations with susceptibility and phenotype expression in sarcoidosis.

Morais A(1), Lima B, Peixoto MJ, Alves H, Marques A, Delgado L.

4.

Invest Ophthalmol Vis Sci. 2012 Oct 11;53(11):7109-15. doi:10.1167/iovs.12-10491.

Genetic characterization and susceptibility for sarcoidosis in Japanese patients: risk factors of BTNL2 gene polymorphisms and HLA class II alleles.

5.

Rev Mal Respir. 2011 Apr;28(4):409-18. doi: 10.1016/j.rmr.2010.09.035. Epub 2011 Mar 16.

[Sarcoidosis and genetics].

Pacheco Y.

6.

Clin Respir J. 2011 Apr;5(2):105-11. doi: 10.1111/j.1752-699X.2010.00206.x.

The BTNL2 A allele variant is frequent in Danish patients with sarcoidosis.

Milman N(1), Svendsen CB, Nielsen FC, van Overeem Hansen T.

7.

Hum Immunol. 2011 Apr;72(4):342-7. doi: 10.1016/j.humimm.2011.01.011. Epub 2011 Jan 20.

Butyrophilin-like 2 in pulmonary sarcoidosis: a factor for susceptibility and progression?

Wijnen PA(1), Voorter CE, Nelemans PJ, Verschakelen JA, Bekers O, Drent M.

8.

Rev Med Interne. 2011 Feb;32(2):73-9. doi: 10.1016/j.revmed.2010.09.011. Epub 2010 Nov 9.

[Pathogenesis of sarcoidosis].

Pacheco Y

9.

Sarcoidosis Vasc Diffuse Lung Dis. 2009 Jul;26(2):162-6.

Homozygous variant rs2076530 of BTNL2 and familial sarcoidosis.

Coudurier M(1), Freymond N, Aissaoui S, Calender A, Pacheco Y, Devouassoux G.

10.

Infect Genet Evol. 2010 May;10(4):517-21. doi: 10.1016/j.meegid.2010.02.006. Epub 2010 Feb 20.

Analysis of the association between BTNL2 polymorphism and tuberculosis in Chinese Han population.

Lian Y(1), Yue J, Han M, Liu J, Liu L

11.

Tissue Antigens. 2009 Jan;73(1):59-61. doi: 10.1111/j.1399-0039.2008.01180.x.

Extended genetic analysis of BTNL2 in sarcoidosis.

Li Y(1), Pabst S, Lokhande S, Grohé C, Wollnik B.

12.

Allergol Int. 2009 Mar;58(1):29-35. doi: 10.2332/allergolint.08-OA-0005. Epub 2008 Dec 1.

Genetic impact of a butyrophilin-like 2 (BTNL2) gene variation on specific IgE responsiveness to Dermatophagoides farinae (Der f) in Japanese.

Konno S(1), Takahashi D, Hizawa N, Hattori T, Takahashi A, Isada A, Maeda Y, Huang SK, Nishimura M.

13.

Curr Opin Pulm Med. 2008 Sep;14(5):434-9. doi: 10.1097/MCP.0b013e3283043de7.

Genetics of sarcoidosis.

Grunewald J.

14.

Respiration. 2007;74(6):601-8.

Genetic predisposition to respiratory diseases: infiltrative lung diseases.

Steele MP(1), Brown KK.

15.

Cardiology. 2008;109(2):117-21. Epub 2007 Aug 17.

Isolated cardiac sarcoidosis associated with the expression of a splice variant coding for a truncated BTNL2 protein.

Meyer T(1), Lauschke J, Ruppert V, Richter A, Pankuweit S, Maisch B.

16.

Proc Am Thorac Soc. 2007 Aug 15;4(5):457-60.

Advances in the genetics of sarcoidosis.

Iannuzzi MC.

17.

Tissue Antigens. 2007 Sep;70(3):219-27.

Analysis of BTNL2 genetic polymorphisms in British and Dutch patients with sarcoidosis.

Spagnolo P(1), Sato H, Grutters JC, Renzoni EA, Marshall SE, Ruven HJ, Wells AU,

Tzouvelekis A, van Moorsel CH, van den Bosch JM, du Bois RM, Welsh KI

18.

Tissue Antigens. 2007 Aug;70(2):128-35.

Butyrophilin-like 2 gene is associated with ulcerative colitis in the Japanese under strong linkage disequilibrium with HLA-DRB1*1502.

Mochida A(1), Kinouchi Y, Negoro K, Takahashi S, Takagi S, Nomura E, Kakuta Y,

Tosa M, Shimosegawa T

19.

Clin Dermatol. 2007 May-Jun;25(3):242-9.

Genetics of sarcoidosis.

Spagnolo P(1), du Bois RM.

20.

Tissue Antigens. 2007 Mar;69(3):236-41.

Analysis of the BTNL2 truncating splice site mutation in tuberculosis, leprosy and Crohn's disease.

Johnson CM(1), Traherne JA, Jamieson SE, Tremelling M, Bingham S, Parkes M, Blackwell JM, Trowsdale J.

21.

Semin Respir Crit Care Med. 2007 Feb;28(1):15-21.

Genetics of sarcoidosis.

Iannuzzi MC.

22.

Am J Respir Crit Care Med. 2007 Mar 1;175(5):498-506. Epub 2006 Dec 14.

Expression of receptor for advanced glycation end products in sarcoid granulomas.

Campo I(1), Morbini P, Zorzetto M, Tinelli C, Brunetta E, Villa C, Bombieri C,Cuccia M, Agostini C, Bozzi V, Facoetti A, Ferrarotti I, Mazzola P, Scabini R,Semenzato G, Pignatti PF, Pozzi E, Luisetti M.

23. Clin Endocrinol (Oxf). 2006 Oct;65(4):429-32.

Association of the BTNL2 rs2076530 single nucleotide polymorphism with Graves' disease appears to be secondary to DRB1 exon 2 position beta74.

Simmonds MJ(1), Heward JM, Barrett JC, Franklyn JA, Gough SC.

24.

J Immunol. 2006 Jun 15;176(12):7354-60.

BTNL2, a butyrophilin-like molecule that functions to inhibit T cell activation.

Nguyen T(1), Liu XK, Zhang Y, Dong C.

25.

BMC Med Genet. 2006 Mar 9;7:21.

On the Wegener granulomatosis associated region on chromosome 6p21.3.

Szyld P(1), Jagiello P, Csernok E, Gross WL, Epplen JT.

26. Thorax. 2006 Mar;61(3):273-4.

BTNL2 gene variant and sarcoidosis.

Li Y, Wollnik B, Pabst S, Lennarz M, Rohmann E, Gillissen A, Vetter H, Grohé C.

27.

Hum Mol Genet. 2006 Jan 1;15(1):155-61. Epub 2005 Dec 1.

Association of the truncating splice site mutation in BTNL2 with multiple sclerosis is secondary to HLA-DRB1*15.

Traherne JA(1), Barcellos LF, Sawcer SJ, Compston A, Ramsay PP, Hauser SL, Oksenberg JR, Trowsdale J.

28.

Am J Hum Genet. 2005 Sep;77(3):491-9. Epub 2005 Jul 20.

The BTNL2 gene and sarcoidosis susceptibility in African Americans and Whites.

Rybicki BA(1), Walewski JL, Maliarik MJ, Kian H, Iannuzzi MC; ACCESS Research Group.

29.

Nat Genet. 2005 Apr;37(4):357-64. Epub 2005 Feb 27.

Sarcoidosis is associated with a truncating splice site mutation in BTNL2.

Valentonyte R(1), Hampe J, Huse K, Rosenstiel P, Albrecht M, Stenzel A, Nagy M, Gaede KI, Franke A, Haesler R, Koch A, Lengauer T, Seegert D, Reiling N, Ehlers S, Schwinger E, Platzer M, Krawczak M, Müller-Quernheim J, Schürmann M, Schreiber S.

30.

Genome-wide association study of African and European Americans implicates multiple shared and ethnic specific loci in sarcoidosis susceptibility.

Adrianto I., Lin C.P., Hale J.J., Levin A.M., Datta I., Parker R., Adler A., Kelly J.A., Kaufman K.M., Lessard C.J., Moser K.L., Kimberly R.P., Harley J.B., Iannuzzi M.C., Rybicki B.A., Montgomery C.G.

PLoS ONE. 7 (8) , 2012. Article Number: e43907. Date of Publication: 27 Aug 2012.

31.

Erratum: Sarcoidosis is associated with a truncating splice site mutation in the gene BTNL2 (Nature Genetics (2005) 37 (357-364)).

Valentonyte R., Hampe J., Huse K., Rosenstiel P., Albrecht M., Stenzel A., Nagy M., Gaede K.I., Franke A., Haesler R., Koch A., Lengauer T., Seegert D., Reiling N., Ehlers S., Schwinger E., Platzer M., Krawczak M., Muller-Quernheim J., Schurmann M., Schreiber S.

Nature Genetics. 37 (6) (pp 652), 2005. Date of Publication: June 2005.

32.

Allelic variation in BTNL2 and susceptibility to tuberculosis in a South African population.

Moller M., Kwiatkowski R., Nebel A., van Helden P.D., Hoal E.G., Schreiber S.

Microbes and Infection. 9 (4) (pp 522-528), 2007. Date of Publication: April 2007.

**Records excluded for :**

***Non-association studies**

***Association studies for other diseases**

***Association studies for other polymorphisms**

**n = 40**

1.

BMC Genet. 2013 Jul 6;14:61. doi: 10.1186/1471-2156-14-61.

A common 56-kilobase deletion in a primate-specific segmental duplication creates a novel butyrophilin-like protein.

Aigner J(1), Villatoro S, Rabionet R, Roquer J, Jiménez-Conde J, Martí E, Estivill X.

2.

Hum Genet. 2013 Jul;132(7):803-10. doi: 10.1007/s00439-013-1292-5. Epub 2013 Mar 31.

Replication of genetic loci for sarcoidosis in US black women: data from the Black Women's Health Study.

Cozier Y(1), Ruiz-Narvaez E, McKinnon C, Berman J, Rosenberg L, Palmer J.

3.

Rev Mal Respir. 2011 Apr;28(4):409-18. doi: 10.1016/j.rmr.2010.09.035. Epub 2011 Mar 16.

[Sarcoidosis and genetics].

Pacheco Y.

4.

Rev Med Interne. 2011 Feb;32(2):73-9. doi: 10.1016/j.revmed.2010.09.011. Epub 2010 Nov 9.

[Pathogenesis of sarcoidosis].

Pacheco Y

5.

Sarcoidosis Vasc Diffuse Lung Dis. 2009 Jul;26(2):162-6.

Homozygous variant rs2076530 of BTNL2 and familial sarcoidosis.

Coudurier M(1), Freymond N, Aissaoui S, Calender A, Pacheco Y, Devouassoux G.

6.

Infect Genet Evol. 2010 May;10(4):517-21. doi: 10.1016/j.meegid.2010.02.006. Epub 2010 Feb 20.

Analysis of the association between BTNL2 polymorphism and tuberculosis in Chinese Han population.

Lian Y(1), Yue J, Han M, Liu J, Liu L

7.

Pneumologie. 2009 Mar;63(3):166-75.

[Genetics of sarcoidosis: a key to understanding its pathogenesis].

Müller-Quernheim J(1), Schürmann M, Hofmann S, Gaede KI, Fischer A, Prasse A,

Zissel G, Schreiber S.

8.

Tissue Antigens. 2009 Jan;73(1):59-61. doi: 10.1111/j.1399-0039.2008.01180.x.

Extended genetic analysis of BTNL2 in sarcoidosis.

Li Y(1), Pabst S, Lokhande S, Grohé C, Wollnik B.

9.

Allergol Int. 2009 Mar;58(1):29-35. doi: 10.2332/allergolint.08-OA-0005. Epub 2008 Dec 1.

Genetic impact of a butyrophilin-like 2 (BTNL2) gene variation on specific IgE responsiveness to Dermatophagoides farinae (Der f) in Japanese.

Konno S(1), Takahashi D, Hizawa N, Hattori T, Takahashi A, Isada A, Maeda Y, Huang SK, Nishimura M.

10.

Curr Opin Pulm Med. 2008 Sep;14(5):434-9. doi: 10.1097/MCP.0b013e3283043de7.

Genetics of sarcoidosis.

Grunewald J.

11.

Respiration. 2007;74(6):601-8.

Genetic predisposition to respiratory diseases: infiltrative lung diseases.

Steele MP(1), Brown KK.

12.

Cardiology. 2008;109(2):117-21. Epub 2007 Aug 17.

Isolated cardiac sarcoidosis associated with the expression of a splice variant coding for a truncated BTNL2 protein.

Meyer T(1), Lauschke J, Ruppert V, Richter A, Pankuweit S, Maisch B.

13.

Proc Am Thorac Soc. 2007 Aug 15;4(5):457-60.

Advances in the genetics of sarcoidosis.

Iannuzzi MC.

14.

Tissue Antigens. 2007 Aug;70(2):128-35.

Butyrophilin-like 2 gene is associated with ulcerative colitis in the Japanese under strong linkage disequilibrium with HLA-DRB1*1502.

Mochida A(1), Kinouchi Y, Negoro K, Takahashi S, Takagi S, Nomura E, Kakuta Y,

Tosa M, Shimosegawa T

15.

Clin Dermatol. 2007 May-Jun;25(3):242-9.

Genetics of sarcoidosis.

Spagnolo P(1), du Bois RM.

16.

Tissue Antigens. 2007 Mar;69(3):236-41.

Analysis of the BTNL2 truncating splice site mutation in tuberculosis, leprosy and Crohn's disease.

Johnson CM(1), Traherne JA, Jamieson SE, Tremelling M, Bingham S, Parkes M, Blackwell JM, Trowsdale J.

17.

Microbes Infect. 2007 Apr;9(4):522-8. Epub 2007 Jan 27.

Allelic variation in BTNL2 and susceptibility to tuberculosis in a South African population.

Möller M(1), Kwiatkowski R, Nebel A, van Helden PD, Hoal EG, Schreiber S.

18.

Semin Respir Crit Care Med. 2007 Feb;28(1):15-21.

Genetics of sarcoidosis.

Iannuzzi MC.

19.

Am J Respir Crit Care Med. 2007 Mar 1;175(5):498-506. Epub 2006 Dec 14.

Expression of receptor for advanced glycation end products in sarcoid granulomas.

Campo I(1), Morbini P, Zorzetto M, Tinelli C, Brunetta E, Villa C, Bombieri C,Cuccia M, Agostini C, Bozzi V, Facoetti A, Ferrarotti I, Mazzola P, Scabini R,Semenzato G, Pignatti PF, Pozzi E, Luisetti M.

20.

Clin Endocrinol (Oxf). 2006 Oct;65(4):429-32.

Association of the BTNL2 rs2076530 single nucleotide polymorphism with Graves' disease appears to be secondary to DRB1 exon 2 position beta74.

Simmonds MJ(1), Heward JM, Barrett JC, Franklyn JA, Gough SC.

21.

J Immunol. 2006 Jun 15;176(12):7354-60.

BTNL2, a butyrophilin-like molecule that functions to inhibit T cell activation.

Nguyen T(1), Liu XK, Zhang Y, Dong C.

22.

BMC Med Genet. 2006 Mar 9;7:21.

On the Wegener granulomatosis associated region on chromosome 6p21.3.

Szyld P(1), Jagiello P, Csernok E, Gross WL, Epplen JT.

23.

Hum Mol Genet. 2006 Jan 1;15(1):155-61. Epub 2005 Dec 1.

Association of the truncating splice site mutation in BTNL2 with multiple sclerosis is secondary to HLA-DRB1*15.

Traherne JA(1), Barcellos LF, Sawcer SJ, Compston A, Ramsay PP, Hauser SL, Oksenberg JR, Trowsdale J.

24.

Sarcoidosis and uveitis.

Jamilloux Y., Kodjikian L., Broussolle C., Seve P.

Autoimmunity Reviews. 13 (8) (pp 840-849), 2014. Date of Publication: August 2014.

25.

Characterization of the lung microbiome in sarcoidosis and healthy individuals with respect to host genotype.

Zimmermann A., Hasler R., Zissel G., Gaede K.I., Muller-Quernheim J., Prasse A., Fischer A., Nebel A., Schreiber S.

Pneumologie. Conference: Chronic Inflammatory Disorders of the Lung 2012 Freiburg Germany. Conference Start: 20120928 Conference End: 20120929. Conference Publication: (var.pagings).

26.

Novel MHC associations in sarcoidosis.

Wennerstrom A., Pietinalho A., Salli K., Lasota J., Selroos O., Lokki M.-L.

Tissue Antigens. Conference: 27th EFI European Immunogenetics and Histocompatibility Conference Maastricht Netherlands. Conference Start: 20130511 Conference End: 20130514. Conference Publication: (var.pagings). 81 (5) (pp 375), 2013. Date of Publication: May 2013.

27.

Selected non-MHC variants and acute graft vs host disease after allogeneic haematopoietic stem cell transplantation.

Mrazek F., Kriegova E., Ambruzova Z., Staffova K., Stahelova A., Raida L., Indrak K., Petrek M.

Tissue Antigens. Conference: 27th EFI European Immunogenetics and Histocompatibility Conference Maastricht Netherlands. Conference Start: 20130511 Conference End: 20130514. Conference Publication: (var.pagings). 81 (5) (pp 317-318), 2013. Date of Publication: May 2013.

28.

Evaluation of BTNL2 gene mutation in rs2076530 allele in patients with the diagnosis of skin sarcoidosis.

Hesari K.K., Montaser-Kouhsari L., Taleb S., Hashemi M.A., Rahimpour S., Firooz A.

American Journal of Dermatopathology. Conference: 31st Symposium of the ISDP Barcelona Spain. Conference Start: 20101021 Conference End: 20101023. Conference Publication: (var.pagings). 33 (2) (pp 208), 2011. Date of Publication: April 2011.

29.

Genetic predisposition to cutaneous sarcoidosis.

Shahkarami S., Rezaei N.

Acta Medica Iranica. 50 (10) (pp 721-722), 2012. Date of Publication: 2012.

30.

A genome-wide association study identifies new candidate loci associated with sarcoidosis in european americans.

Adrianto I., Lin C.P., Lessard C.J., Datta I., Levin A.M., Parker R., Adler A., Kaufman K.M., Moser K.L., Iannuzzi M.C., Rybicki B.A., Montgomery C.G.

American Journal of Respiratory and Critical Care Medicine. Conference: American Thoracic Society International Conference, ATS 2011 Denver, CO United States. Conference Start: 20110513 Conference End: 20110518. Conference Publication: (var.pagings). 183 (1 MeetingAbstracts) , 2011. Date of Publication: 01 May 2011.

31.

Genome-wide association study of African Americans implicates multiple lung and inflammatory disease-associated loci in sarcoidosis susceptibility.

Adrianto I., Lin C.P., Hale J.J., Levin A.M., Datta I., Parker R., Adler A.

Arthritis and Rheumatism. Conference: Annual Scientific Meeting of the American College of Rheumatology and Association of Rheumatology Health Professionals 2011 Chicago, IL United States. Conference Start: 20111104 Conference End: 20111109. Conference Publication: (var.pagings). 63 (10 SUPPL. 1) , 2011. Date of Publication: October 2011.

32.

Butyrophilin-like 2 and HLA-DRB1*15: Markers for susceptibility and progression in pulmonary sarcoidosis.

Voorter C.E.M., Wijnen P.A., Nelemans P.J., Verschakelen J.A., Bekers O., Drent M.

Tissue Antigens. Conference: 25th European Immunogenetics and Histocompatibility Conference Prague Czech Republic. Conference Start: 20110504 Conference End: 20110507. Conference Publication: (var.pagings). 77 (5) (pp 395), 2011. Date of Publication: May 2011.

33.

Rheumatic manifestations of systemic disease: Sarcoidosis.

Chatham W.

Current Opinion in Rheumatology. 22 (1) (pp 85-90), 2010. Date of Publication: January 2010.

34.

BTNL2 polymorphism and cardiac sarcoidosis.

Meyer T., Lauschke J., Ruppert V., Richter A., Pankuweit S., Maisch B.

Cardiology. 112 (1) (pp 78-79), 2009. Date of Publication: November 2008.

35.

Cardiac sarcoidosis associated with BTNL2.

Becker C.D., Sridhar P., Iannuzzi M.C.

Cardiology. 112 (1) (pp 76-77), 2009. Date of Publication: November 2008.

36.

Prediction of disease-associated single nucleotide polymorphisms using virtual genomes constructed from a public haplotype database.

Toyabe S.-I., Miyashita A., Kitamura N., Kuwano R., Akazawa K.

Methods of Information in Medicine. 47 (6) (pp 522-528), 2008. Date of Publication: 2008.

37.

Cosignaling complexity gets more convoluted: The emerging importance of the B7-like butyrophilin family of immune regulators.

Arnett H.A., Swanson R.M., Viney J.L.

Current Immunology Reviews. 4 (1) (pp 43-52), 2008. Date of Publication: February 2008.

38.

BTNL2 allele associations with chronic beryllium disease in HLA-DPB1*Glu69-negative individuals.

Sato H., Spagnolo P., Silveira L., Welsh K.I., Du Bois R.M., Newman L.S., Maier L.A.

Tissue Antigens. 70 (6) (pp 480-486), 2007. Date of Publication: December 2007.

39.

Allelic variation in BTNL2 and susceptibility to tuberculosis in a South African population.

Moller M., Kwiatkowski R., Nebel A., van Helden P.D., Hoal E.G., Schreiber S.

Microbes and Infection. 9 (4) (pp 522-528), 2007. Date of Publication: April 2007.

40.

BTNL2, a butyrophilin/B7-like molecule, is a negative costimulatory molecule modulated in intestinal inflammation.

Arnett H.A., Escobar S.S., Gonzalez-Suarez E., Budelsky A.L., Steffen L.A., Boiani N., Zhang M., Siu G., Brewer A.W., Viney J.L.

Journal of Immunology. 178 (3) (pp 1523-1533), 2007. Date of Publication: 01 Feb 2007.

**2 articles were excluded for no enough data:**

**(1):** Major histocompatibility complex class II and BTNL2 associations in sarcoidosis.

Wennerstrom A., Pietinalho A., Lasota J., Salli K., Surakka I., Seppanen M., Selroos O., Lokki M.-L.

European Respiratory Journal. 42 (2) (pp 550-553), 2013. Date of Publication: 01 Aug 2013.

AN: 2013524768

**(2):**

Genome-wide association study of African and European Americans implicates multiple shared and ethnic specific loci in sarcoidosis susceptibility.

Adrianto I., Lin C.P., Hale J.J., Levin A.M., Datta I., Parker R., Adler A., Kelly J.A., Kaufman K.M., Lessard C.J., Moser K.L., Kimberly R.P., Harley J.B., Iannuzzi M.C., Rybicki B.A., Montgomery C.G.

PloS one. 7 (8) , 2012. Article Number: e43907. Date of Publication: 2012.

**10 articles meeting all criteria:**

1.

Sarcoidosis Vasc Diffuse Lung Dis. 2014 Jul 8;31(2):136-41.

Lack of correlation of BTNL2 polymorphism and cancer risk in sarcoidosis. BTNL2 and cancer risk in sarcoidosis.

Gaillot-Drevon M(1), Calender A, Blay JY, Valeyre D, Israel-Biet D, Roy P,Pacheco Y.

2.

Respir Med. 2012 Dec;106(12):1771-7. doi: 10.1016/j.rmed.2012.08.009. Epub 2012 Sep 25.

BTNL2 gene polymorphism associations with susceptibility and phenotype expression in sarcoidosis.

Morais A(1), Lima B, Peixoto MJ, Alves H, Marques A, Delgado L.

3.

Invest Ophthalmol Vis Sci. 2012 Oct 11;53(11):7109-15. doi:10.1167/iovs.12-10491.

Genetic characterization and susceptibility for sarcoidosis in Japanese patients: risk factors of BTNL2 gene polymorphisms and HLA class II alleles.

4.

Clin Respir J. 2011 Apr;5(2):105-11. doi: 10.1111/j.1752-699X.2010.00206.x.

The BTNL2 A allele variant is frequent in Danish patients with sarcoidosis.

Milman N(1), Svendsen CB, Nielsen FC, van Overeem Hansen T.

5.

Hum Immunol. 2011 Apr;72(4):342-7. doi: 10.1016/j.humimm.2011.01.011. Epub 2011 Jan 20.

Butyrophilin-like 2 in pulmonary sarcoidosis: a factor for susceptibility and progression?

Wijnen PA(1), Voorter CE, Nelemans PJ, Verschakelen JA, Bekers O, Drent M.

6.

Tissue Antigens. 2007 Sep;70(3):219-27.

Analysis of BTNL2 genetic polymorphisms in British and Dutch patients with sarcoidosis.

Spagnolo P(1), Sato H, Grutters JC, Renzoni EA, Marshall SE, Ruven HJ, Wells AU,

Tzouvelekis A, van Moorsel CH, van den Bosch JM, du Bois RM, Welsh KI

7.

Thorax. 2006 Mar;61(3):273-4.

BTNL2 gene variant and sarcoidosis.

Li Y, Wollnik B, Pabst S, Lennarz M, Rohmann E, Gillissen A, Vetter H, Grohé C.

8.

Am J Hum Genet. 2005 Sep;77(3):491-9. Epub 2005 Jul 20.

The BTNL2 gene and sarcoidosis susceptibility in African Americans and Whites.

Rybicki BA(1), Walewski JL, Maliarik MJ, Kian H, Iannuzzi MC; ACCESS Research Group.

9.

Nat Genet. 2005 Apr;37(4):357-64. Epub 2005 Feb 27.

Sarcoidosis is associated with a truncating splice site mutation in BTNL2.

Valentonyte R(1), Hampe J, Huse K, Rosenstiel P, Albrecht M, Stenzel A, Nagy M, Gaede KI, Franke A, Haesler R, Koch A, Lengauer T, Seegert D, Reiling N, Ehlers S, Schwinger E, Platzer M, Krawczak M, Müller-Quernheim J, Schürmann M, Schreiber S.

10.

Is there a genetic predisposition for Turkish patients with sarcoidosis in the 329-bp region containing the BTNL2 rs2076530 polymorphism?.

Ozdemir M., Saydam F., Kurt E., Degirmenci I., Tuncel T., Cilingir O., Gunes H.V., Artan S.

Turkish Journal of Medical Sciences. 44 (4) (pp 590-594), 2014. Date of Publication: 2014.
